# Veterinary drug therapies used for undesirable behaviours in UK dogs under primary veterinary care

**DOI:** 10.1371/journal.pone.0261139

**Published:** 2022-01-12

**Authors:** Annabel J. Craven, Camilla Pegram, Rowena M. A. Packer, Susan Jarvis, Paul D. McGreevy, Caroline Warnes, David B. Church, Dave C. Brodbelt, Dan G. O’Neill

**Affiliations:** 1 Royal (Dick) School of Veterinary Studies, University of Edinburgh, Easter Bush, United Kingdom; 2 Pathobiology and Population Sciences, The Royal Veterinary College, Hawkshead Lane, North Mymms, Hatfield, Herts, United Kingdom; 3 Clinical Sciences and Services, The Royal Veterinary College, Hawkshead Lane, North Mymms, Hatfield, Herts, United Kingdom; 4 Global Academy of Agriculture and Food Security, Royal (Dick) School of Veterinary Studies, University of Edinburgh, Easter Bush, United Kingdom; 5 School of Environmental and Rural Science, Faculty of Science, Agriculture, Business and Law, University of New England, Armidale, Australia; 6 Independent Researcher, North Devon, United Kingdom; Universidade do Porto Instituto de Biologia Molecular e Celular, PORTUGAL

## Abstract

Undesirable behaviours (UBs) in dogs are common and important issues with serious potential welfare consequences for both the dogs and their owners. This study aimed to investigate the usage of drug therapy for UBs in dogs and assess demographic risk factors for drug-prescribed UBs within the dog population under primary-care veterinary care in the UK in 2013. Dogs receiving drug therapy for UB were identified through the retrospective analysis of anonymised electronic patient records in VetCompass™. Risk factor analysis used multivariable logistic regression modelling. The study population comprised 103,597 dogs under veterinary care in the UK during 2013. There were 413 drug-prescribed UBs recorded among 404 dogs. The prevalence of dogs with at least one UB event treated with a drug in 2013 was 0.4%. Multivariable modelling identified 3 breeds with increased odds of drug-prescribed UB compared with crossbred dogs: Toy Poodle (OR 2.75), Tibetan Terrier (OR 2.68) and Shih-tzu (OR 1.95). Increasing age was associated with increased odds of drug-prescribed UB, with dogs ≥ 12 years showing 3.1 times the odds compared with dogs < 3 years. Neutered males (OR 1.82) and entire males (OR 1.50) had increased odds compared with entire females. The relatively low prevalence of dogs with at least one UB event that was treated with a drug in 2013 could suggest that opportunities for useful psychopharmaceutical intervention in UBs may be being missed in first opinion veterinary practice. While bodyweight was not a significant factor, the 3 individual breeds at higher odds of an UB treated with a behaviour modifying drug all have a relatively low average bodyweight. The current results also support previous research of a male predisposition to UBs and it is possible that this higher risk resulted in the increased likelihood of being prescribed a behaviour modifying drug, regardless of neuter status.

## Introduction

Dogs are popular pets in many countries, including the UK, where an estimated 30–31% of households own a dog [1, 2]. Undesirable behaviours (UBs) in domestic dogs are commonly reported [3–5]. A UK survey of 364 owners reported 97.2% of dogs with current or historic UBs [5], while another study of 192 owners reported that 98% of dogs performed UBs [6]. Many studies report that individual dogs often display more than one UB [7–10]. A US study reported a mean of 1.6 UB diagnoses per referred dog, with 26% exhibiting two UBs, and 18.6% exhibiting three or more UBs [9].

In previous studies of UBs in veterinary practice, UBs have been defined as any behavioural attribute that was recorded in veterinary clinical notes and which the owner and/or other people deemed to be unwelcome [11]. Aggressive behaviour is the most commonly reported UB in dogs referred to behaviourists [10, 12], accounting for approximately 70% of all behavioural referrals in the United States, Canada and Australia [13]. Referral practice data suggests that separation related behaviours (SRBs) are the second most common UBs in dogs referred to behaviourists [9, 14], accounting for 14.4% of dogs evaluated at the Animal Behavior Clinic (Cornell University) between 1991 and 2001 [9]. This is likely an underestimate in comparison to the general owned dog population, with many SRBs not formally diagnosed by a veterinarian, as in owner-reported studies, 34% of UK owners reported signs of SRB in their dogs [6].

Primary practice veterinary clinical notes are recorded with the main purpose of summarising the clinical case and its management and therefore do not aim or need to provide a full account of every aspect of the case. Consequently, research using primary-care veterinary clinical notes may underestimate the true UB prevalence in dogs. Furthermore, the results reported from research using primary-care veterinary clinical notes are likely to differ from results reported from research based on owner surveys and from research based on records recorded for dogs referred to behaviourists. Differences can be observed between the most common UB referred to behaviourists (aggressive behaviour and SRB) [9, 10, 12, 14] and the most common UB reported by owners, attention seeking behaviour [6]. This disparity likely reflects both the real or perceived risk to the dog or humans by primary care veterinarians and owner tolerance: owners may tolerate some UBs more than others, only reporting or seeking help for the most problematic to them during a veterinary consultation. For example, a UK questionnaire and interview-based study reported that only 29% of owners with dogs demonstrating behaviours indicative of noise fears sought any help for them, with just 45% of those owners seeking help sourcing it from their veterinarian [15]. The threshold for considering a behaviour as undesirable likely depends on human perceptions of the behaviour and expectations of what acceptable canine behaviour includes [16].

UBs have a variety of aetiologies, including stress-coping mechanisms, responses to somatic conditions (e.g., polyuria relates to polydipsia with multiple potential causes), and behavioural pathologies such as abnormal repetitive behaviours [17, 18]. Many UBs, such as SRB, indicate compromised welfare and therefore changing the negative emotional states that underlie these behaviours is a clear priority [19]. Meanwhile, the consequences of UBs can be profound for both dogs and owners [20] especially for behaviours that commonly lead to relinquishment and euthanasia [21]. A retrospective analysis of records from a US shelter found behavioural issues (36.1%) and incompatibility with existing pets (18.3%) were the most common reasons for the return of dogs to the shelter [22]. A US study reported that 29.5% of relinquishing owners stipulated a non-aggressive UB as the reason for relinquishment [23] but aggression is reported as the chief precipitant for euthanasia in UK dogs under 3 years of age [11]. UK studies report UBs as the most common cause of death of dogs under the age of 3 years attending primary-care practice. One study reported that 33.7% of dogs died as a result of an UB (including road traffic accidents (RTAs), as RTAs may result from poor recall, limited traffic training or straying) [11], where another study reported 14.7% of deaths ascribed to behavioural abnormality and a further 12.7% ascribed to RTAs (a combined total of 27.4%) [24]. Comparable findings using a similar study design have recently emerged from Australia (29.7%) [25].

**Fig 1:**
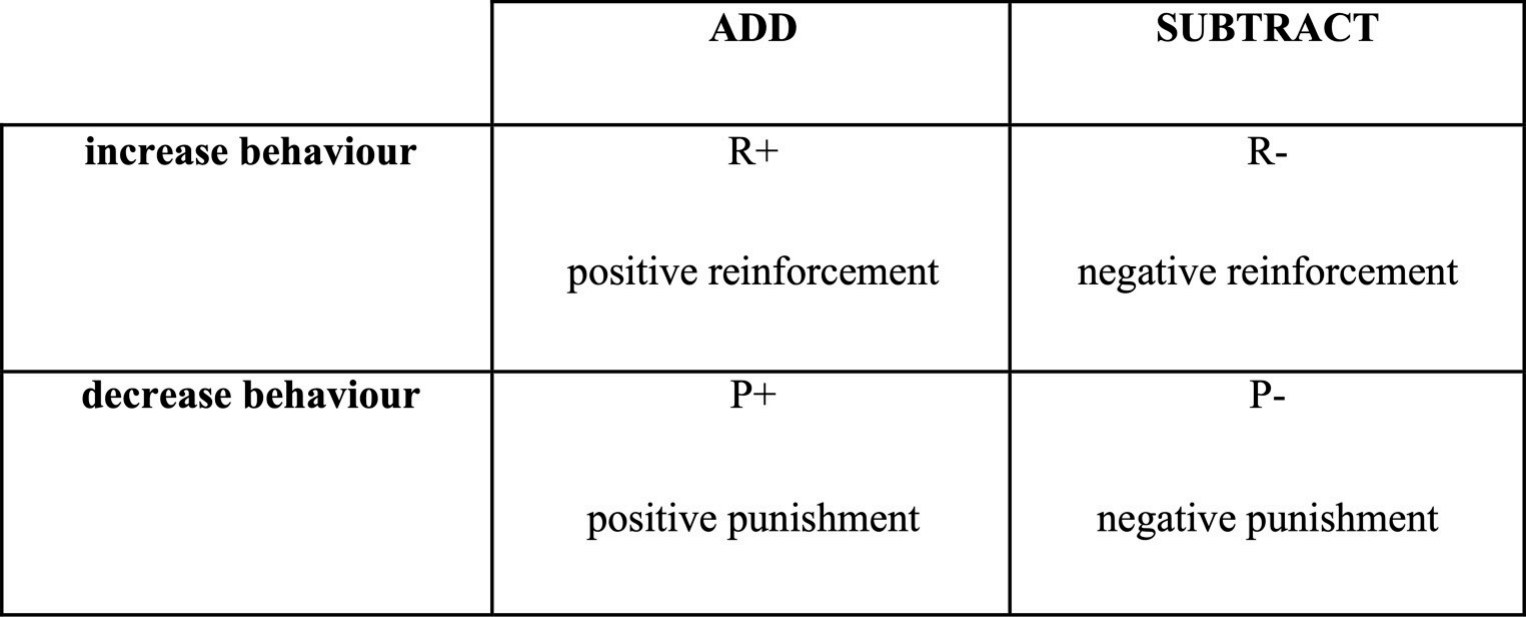
The operant conditioning quadrant. There are four types of operant learning. Two of the quadrants increase the recurrence of a behaviour and are referred to as reinforcement. The other two quadrants decrease the recurrence of a behaviour and are referred to as punishment. The terms negative and positive indicate whether a stimulus has been subtracted (negative) or added (positive) to result in an increase or decrease of a behaviour.

Veterinary clinical management of behavioural cases often encompasses control of the environment (to reduce behavioural triggers and enrich the dog’s social and physical surroundings), behavioural modification (including training and improved dogmanship) and pharmaceutical support. Given that some UBs may have become habitual and self-reinforcing, they may be exhibited by dogs over weeks and months before reaching a level of undesirability that precipitates them being addressed by owners [26]. This partly explains why quick fixes in behavioural modification are rare. The roles of affective (emotional) state, arousal and attachment (the so-called ‘3As’) in any operant conditioning challenge are now recognised [27]. The 3 A’s influence the choice of operant conditioning quadrant (Fig 1) in which to work and increase the focus on good dogmanship [28] rather than blaming only the dog for every shortfall in response to therapy. In some cases, drug therapy may help to decrease arousal in the dog, improve negative emotional states and facilitate behaviour modification [29–31].

A variety of drugs have been used in dogs to treat a range of UBs including aggression, anxiety, ‘compulsive’ behaviours, hyperactivity and cognitive dysfunction [17], although the scientific literature underpinning their use and efficacy is sparse. Clomipramine has been reported to be effective when used in combination with behaviour modification to manage UBs such as noise phobias and compulsive disorders [29]. Studies suggest that benzodiazepines, such as diazepam, can be used to provide an immediate anxiolytic effect while others, such as selective serotonin reuptake inhibitors (SSRI), take weeks to reach therapeutic levels and so have a longer-term role in treating anxiety disorders [30]. Although outcomes of studies using drugs for the treatment of UB are often assessed using owner reports, more nuanced assessments are increasingly being used; for example, there is evidence that SSRIs may not simply help to reduce the UB, but also improve the dog’s affective state [31].

Many contributing factors are reported to influence decision-making on the use of psychopharmaceutical therapy for UBs. They include perceptions by veterinary teams and dog owners of what is ‘normal’ and acceptable canine behaviour as well as issues around cost and safety [32] and the ease of administration [19], which have been reviewed elsewhere (e.g. [33]). However, there is limited published information on the usage of pharmaceutical treatment to manage UBs in dogs under primary veterinary care and it is widely acknowledged that such data on veterinary behavioural psychopharmacology is needed [34, 35]. Consequently, the current study aimed to investigate the one-year period prevalence of UB drug therapy and evaluate demographic risk factors (*purebred*, *breed*, *adult bodyweight*, *age*, *sex-neuter* and *insurance*) for drug therapy used for UBs within the dog population under primary-care veterinary care in the UK. Given reports that increasing bodyweight is negatively correlated with the prevalence of reported UBs [36–38], we hypothesise that dogs with smaller bodyweight have a higher probability of receiving drug therapy aimed at managing UBs than dogs with heavier bodyweight.

## Methods

### VetCompass™

The VetCompass™ Programme collates de-identified electronic patient record (EPR) data from primary-care veterinary practices in the UK for epidemiological research [39]. Shared data include patient background information (species, age, sex, weight, breed, neuter status and insurance status), treatment, deceased status, clinical description terms (The VeNom Coding Group: VeNom Veterinary Nomenclature) and free-text clinical notes. The data collection, collation and extraction methods have been reported previously [39–41].

Sample size calculation estimated that 4,847 dogs of smaller bodyweight (< 10kg) and 19,385 dogs of larger bodyweight (> 10kg) were required to detect an odds ratio of 1.5 times or greater for a drug-prescribed UB, assuming that 1.0% of the heavier dogs had a drug-prescribed UB (4:1 ratio of dogs > 10kg: dogs < 10kg, two-sided 95% confidence interval, 80% power) [1, 42]. Ethical approval was granted by the RVC Ethics and Welfare Committee (reference number SR2018-1652).

### Selection criteria and definitions

The current study defined an undesirable behaviour (UB) as any behaviour pattern with evidence in the clinical records that it was troublesome to at least one human associated with the dog including members of the veterinary team, trainers and groomers as well as the owners, friends or strangers who interacted with the dog. Because information about individual UBs was taken directly from the clinical records, it reflects the primary-care veterinary description or interpretation of the UB, and cannot be assumed to be a precise behavioural diagnosis.

A list of drugs (S1 Table) recommended for use in dogs to treat UBs was developed using information from five key resources [17, 26, 43–45]. The drugs were classified by legal category using the National Office of Animal Health (NOAH) online database [46]. A ‘stem’ term was generated for each drug (e.g. ‘diaze’ for diazepam) (S1 Table). The categories of medications included were: Prescription Only Medicine–Veterinarian (POM-V), Prescription Only Medicine–Controlled Drug (POM-CD) and off-license (S1 Table). General Sales List (GSL) drugs (such as Adaptil^TM^, Ceva Animal Health) were excluded because these can be sold as over-the-counter products without a formally recorded veterinary clinical description of an UB. Phenylpropanolamine (Propalin™) was excluded because this drug is used to treat organic medical conditions (i.e. urinary incontinence caused by incompetence of the urethral sphincter [47]) rather than the urination behaviour itself. Injectable versions of acepromazine maleate, diazepam, midazolam and temazepam were also excluded because of their common use as a pre-medication for surgery. However, any of these drugs with an indication of administration in any other liquid form (such as rectally) were included.

For the purpose of this study, UBs explicitly stemming from underlying organic disease were excluded. Epilepsy and seizure disorders in dogs are commonly treated with drugs that have behaviour modifying activities [45]. For the purposes of the current study, dogs with evidence of epilepsy and seizure disorders were excluded as UBs because epilepsy is considered a neurological, rather than behavioural, condition [48]. Dogs prescribed behaviour modifying drugs for the management of disorders related to dementia were included within the study and were recorded from the Venom list as ‘cognitive dysfunction’. Cognitive dysfunction was included as an UB in the study because many of the primary presenting clinical signs that contributed to this diagnosis, such as vocalising at night, were attributed by the veterinarian to cognitive dysfunction where there was often potential for these UBs to have an alternative behavioural cause. However, if clinical signs included a head tilt or the dog was diagnosed with vestibular disease, the case was excluded as treatment may have been primarily directed at the organic disease rather than the behaviour itself [49]. Cognitive dysfunction was also included as an UB in the study because it is a relatively commonly recorded UB by primary-care practitioners. This study aimed to reflect the common UBs recorded and clinically managed by primary-care in order to give a fuller picture of the wider real-world views on UBs in dogs. Behaviour modifying drugs prescribed to treat pseudopregnancy were included within the study only when specific UBs, such as ‘nesting behaviour’, were recorded in the clinical notes as being undesirable from the owner’s perspective. Pseudopregnant bitches without stated clinical behavioural signs were excluded.

The sampling frame for the current study included all dogs from four practice groups that had at least one electronic patient record (EPR) (clinical note, bodyweight or treatment) recorded in the VetCompass™ database during 2013 or at least one EPR recorded both before and after 2013. Key word searches identified all dogs from the study sampling frame with at least one instance of a drug selected for inclusion (S1 Table) from January 01, 2013 to December 31, 2013.

### Data extraction

The full clinical notes at any date in the available records of dogs with at least one behaviour modifying drug prescribed in 2013 were manually reviewed to extract information on the primary reason recorded for using the medication. If the drug(s) in question appeared to have been prescribed with the clinical aim of directly modifying an UB using drug therapy, the clinical description term for the specific UB(s) was/were extracted by linking to the most appropriate VeNom terms. The extracted clinical description terms were mapped to a dual hierarchy of clinically descriptive precision for analysis: specific terms and grouped terms, as previously described [39]. The study included the most precise clinical description terms as recorded in the clinical notes. In many cases the most precise clinical description may in reality be a clinical sign, such as ‘muzzle for examination as attempts to bite’, which would be recorded as the closest available term, in this case ‘aggressive’ at the grouped level and ‘aggressive for procedure’ at the specific level. Information was extracted on all deaths on any date during the available clinical records to describe the date of death, whether it related to an UB and the mechanism of death (non-assisted or euthanasia).

### Variables

Breed information entered by the participating practices was cleaned and mapped to a VetCompass™ breed list derived and extended from the VeNom Coding breed list [50]. A *purebred* variable categorised all dogs of recognisable breeds as ‘purebred’, ‘designer’ or ‘crossbred’ [51, 52]. A *breed* variable included individual breeds represented by over 1,000 dogs in the overall study or with ≥ 5 drug-prescribed UB cases, a grouped category of all remaining purebreds and a general grouping of crossbred dogs. This approach was adopted to optimise statistical power for the individual breed analyses [53]. *Sex-Neuter status* was defined by the final available EPR neuter value and was combined with sex to create four categories: female entire, female neutered, male entire and male neutered. *Adult bodyweight* for each dog was defined as the mean of all bodyweight (kg) values recorded after 18 months old. *Adult bodyweight* (kg) was categorised as: <10, 10 to < 20, 20 to < 30 and ≥ 30. *Age* defined the age (years) on 31^st^ December 2013 and was categorised: < 3.0, 3.0 to < 6.0, 6.0 to < 9.0, 9.0 to < 12.0 and ≥ 12.0. The individual clinics that were included in this study were part of four large practice groups that were distributed throughout the UK and were assigned a code during analysis to preserve anonymity. *Insurance status* was categorised according to whether the dog was recorded as insured or not insured in the final EPR. Missing data were recorded as “Not recorded” and included as a separate category in the analysis if they accounted for >10% of the study variable, otherwise missing data were excluded [54]. Following data-checking for internal validity and cleaning in Excel (Microsoft Office Excel 2007, Microsoft Corp), analyses were conducted using SPSS version 24.0 (IBM Corp).

### Statistical analysis

Normality of continuous variables was assessed graphically and using the Kolmogorov–Smirnov (K–S) test for normality [55]. All continuous variables were non-normally distributed and so were summarised using median, interquartile range (IQR) and ranges. Chi-square or Fisher’s exact test were used to compare categorical variables and the Mann-Whitney U test was used to compare continuous variables [56]. The one-year period prevalence with 95% confidence intervals (CI) described the probability of at least one drug-prescribed UB being recorded during 2013.

Binary logistic regression modelling was used to evaluate univariable associations between risk factors (*purebred*, *breed*, *adult bodyweight*, *age*, *sex-neuter* and *insurance*) and drug- prescribed UB. Risk factors with liberal associations in univariable modelling (P < 0.2) were taken forward for multivariable evaluation. Collinearity was investigated by examining the variance inflation factor (VIF) and tolerance, with collinearity indicated if VIF > 10 and tolerance < 0.1 [57, 58]. Variables identified as highly collinear with *breed* (*purebred* and *adult bodyweight*) were excluded from the initial *breed* multivariable modelling. To evaluate their effects after taking account of the other variables, each collinear variable that was liberally associated at the univariable stage was used to individually replace the *breed* variable in the main final model [59]. Model development used manual backwards stepwise elimination, whereby the least significant variable was removed at each step until all remaining variables in the model had p-value < 0.05 [60]. Potential confounders were assessed by checking for a marked change (> 10%) in the odds ratio (OR) after removal of the variable from the model [61]. Biologically plausible pairwise interactions in the final model were examined using the Wald test [60]. Practice group attended was evaluated as a fixed effect. The area under the ROC curve and the Hosmer-Lemeshow test were used to evaluate the quality of the model fit [60, 62]. Statistical significance was set at the 5% level.

## Results

### Proportion of dogs with at least one UB event that was treated with a drug

**Fig 2:**
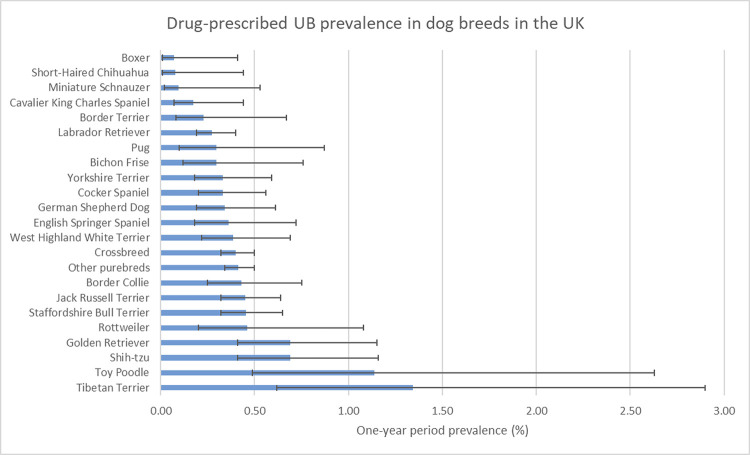
One-year (2013) period prevalence of drug-prescribed UB in commonly affected dog breeds attending primary-care veterinary practices in the VetCompass™ Programme in the UK. The error bars show the 95% confidence interval.

From a study population of 104,212 dogs under veterinary care in the UK, there were 413 drug-prescribed UB events recorded for 404 dogs during 2013. The estimated one-year period prevalence of dogs with at least one UB event that was treated with a drug was 0.4% (95% CI 0.35–0.43). The breeds with the highest drug-prescribed UB prevalence were the Tibetan Terrier (1.3%, 95% CI 0.62–2.90), Toy Poodle (1.1%, 95% CI 0.49–2.63), Shih-tzu (0.7%, 95% CI 0.41–1.16), Golden Retriever (0.7%, 95% CI 0.41–1.15), and Rottweiler (0.5%, 95% CI 0.20–1.08) (Fig 2). Data completeness were: *breed* 99.9%, *age* 99.8%, *sex-neuter status* 99.7%, *insurance status* 100.0% and *adult bodyweight* 75.1%.

### Demography and most common drug-prescribed UBs clinically described

Descriptive analysis included 404 drug-prescribed UB cases and 103,193 non-cases (Table 1). The median age of drug-prescribed UB cases (7.6 years, IQR 3.8–12.3, range 0.9–19.6) was older than for non-cases (5.4 years, IQR 2.6–8.9, range 0.1–21.9) (p < 0.001). The median bodyweight of drug-prescribed UB cases (19.4 kg, IQR 9.8–30.0, range 3.6–70.9) did not differ significantly from non-cases (17.8kg, IQR 9.4–29.3, range 1.2–100.6) (p = 0.162). The most common breeds among drug-prescribed UB cases overall were the Staffordshire Bull Terrier (31; 7.7%), Jack Russell Terrier (30; 7.4%), Labrador Retriever (26; 6.4%), Cocker Spaniel (14; 3.5%), Golden Retriever (14; 3.5%) and Shih-tzu (14; 3.5%), in addition to 78 (19.3%) crossbreds. The most common breeds among non-cases were the Labrador Retriever (9445; 9.2%), Staffordshire bull terrier (6774; 6.6%), Jack Russell terrier (6619; 6.4%), Cocker Spaniel (4202; 4.1%) and Yorkshire Terrier (3326; 3.2%) in addition to 19,503 (18.9%) crossbreds.

**Table 1:** Demography for drug-prescribed UB cases (n = 404) and non-drug-prescribed UB cases (n = 103,193) in dogs attending primary-care veterinary practices in the VetCompass™ Programme in the UK during 2013.

Variable	Category	UB case count (%)	UB non-case count (%)
Breed	Crossbreed	78 (19.3)	19503 (18.9)
	Purebred–Other	110 (27.2)	26562 (25.7)
	Labrador Retriever	26 (6.4)	9445 (9.2)
	Staffordshire Bull Terrier	31 (7.7)	6774 (6.6)
	Jack Russell Terrier	30 (7.4)	6619 (6.4)
	Cocker Spaniel	14 (3.5)	4202 (4.1)
	Yorkshire Terrier	11 (2.7)	3326 (3.2)
	German Shepherd Dog	11 (2.7)	3216 (3.1)
	West Highland White Terrier	11 (2.7)	2838 (2.8)
	Border Collie	12 (3.0)	2766 (2.7)
	Cavalier King Charles Spaniel	4 (1.0)	2310 (2.2)
	English Springer Spaniel	8 (2.0)	2192 (2.1)
	Golden Retriever	14 (3.5)	2017 (2.0)
	Shih-tzu	14 (3.5)	2011 (1.9)
	Boxer	1 (0.2)	1372 (1.3)
	Bichon Frise	4 (1.0)	1337 (1.3)
	Border Terrier	3 (0.7)	1307 (1.3)
	Short-Haired Chihuahua	1 (0.2)	1291 (1.3)
	Rottweiler	5 (1.2)	1077 (1.0)
	Miniature Schnauzer	1 (0.2)	1059 (1.0)
	Pug	3 (0.7)	1005 (1.0)
	Tibetan Terrier	6 (1.5)	441 (0.4)
	Toy Poodle	5 (1.2)	435 (0.4)
	Not recorded	1 (0.2)	88 (0.1)
Purebred	Crossbred	78 (19.3)	19503 (18.9)
	Purebred	315 (78.0)	80041 (77.6)
	Designer	10 (2.5)	3561 (3.5)
	Not recorded	1 (0.2)	88 (0.1)
Bodyweight (kg)	< 10	94 (23.5)	21378 (20.7)
	10 to < 20	95 (23.6)	20966 (20.3)
	20 to < 30	86 (21.3)	16664 (16.1)
	≥ 30	92 (22.8)	18456 (17.9)
	Not recorded	37 (9.2)	25729 (24.9)
Age (years)	< 3	74 (18.3)	29461 (28.5)
	3 to < 6	90 (22.3)	26987 (26.2)
	6 to < 9	69 (17.1)	21080 (20.4)
	9 to < 12	67 (16.6)	14304 (13.9)
	≥ 12	104 (25.7)	11149 (10.8)
	Not recorded	0 (0.0)	212 (0.2)
Sex-Neuter status	Female entire	58 (14.4)	26198 (25.4)
	Female neutered	99 (24.5)	22938 (22.2)
	Male entire	109 (27.0)	30866 (29.9)
	Male neutered	138 (34.2)	22905 (22.2)
	Not recorded	0 (0.0)	286 (0.3)
Insurance	Non–insured	217 (53.7)	71522 (69.3)
	Insured	187 (46.3)	31671 (30.7)
Practice Group	1	337 (83.4)	93642 (90.7)
	2	21 (5.2)	2651 (2.6)
	3	18 (4.5)	3446 (3.3)
	4	28 (6.9)	3454 (3.3)

**Fig 3:**
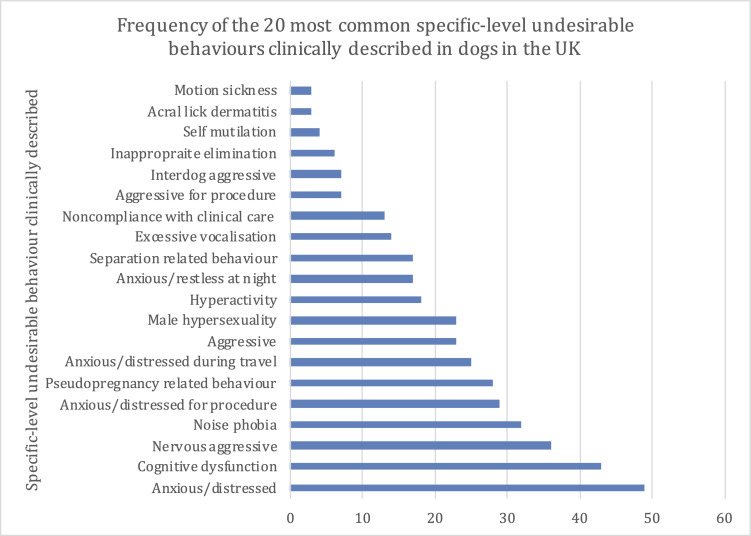
The frequency (count) of the 20 most common specific-level drug-prescribed UBs described in dogs attending primary-care practices in the VetCompass™ Programme in the UK during 2013 (n = 413).

**Fig 4:**
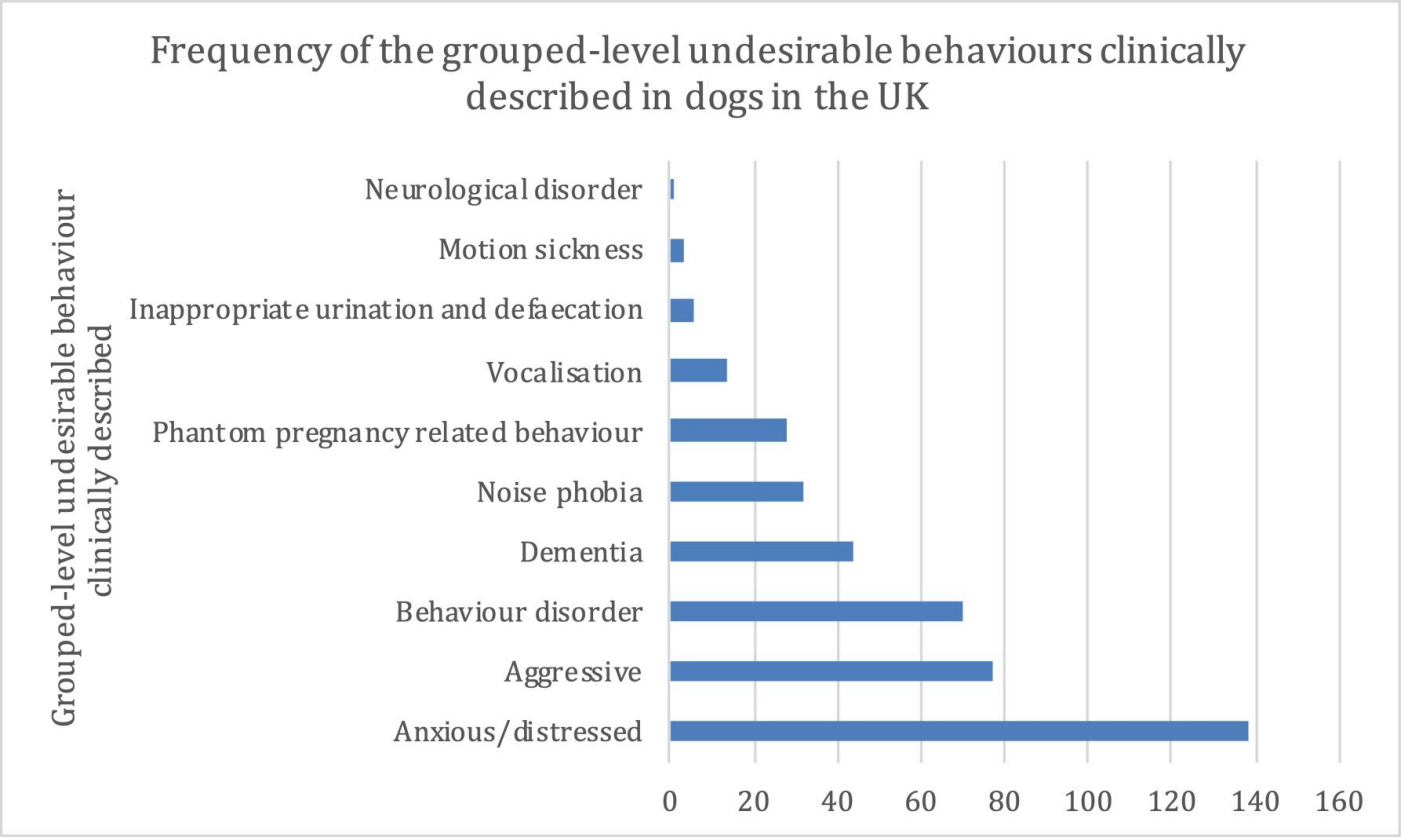
The frequency (count) of the grouped-level drug-prescribed UBs described in dogs attending primary-care practices in the VetCompass™ Programme in the UK during 2013 (n = 413).

There were 413 drug-prescribed UBs recorded for the 404 case dogs. At the most specific-level of clinical description, the most frequent UBs that were prescribed at least one drug were anxious/distressed (11.9%; 49/413), cognitive dysfunction (10.4%; 43) and nervous aggressive (8.7%; 36) (Fig 3). At a more general, grouped-level of clinical description, the most frequent UBs that were associated with a prescription of at least one drug were anxious/distressed (33.4%; 138), aggressive (18.6%; 77) and behaviour disorder (17.0%; 70) (Fig 4).

### Clinical management and outcomes of drug-prescribed UB cases

**Fig 5:**
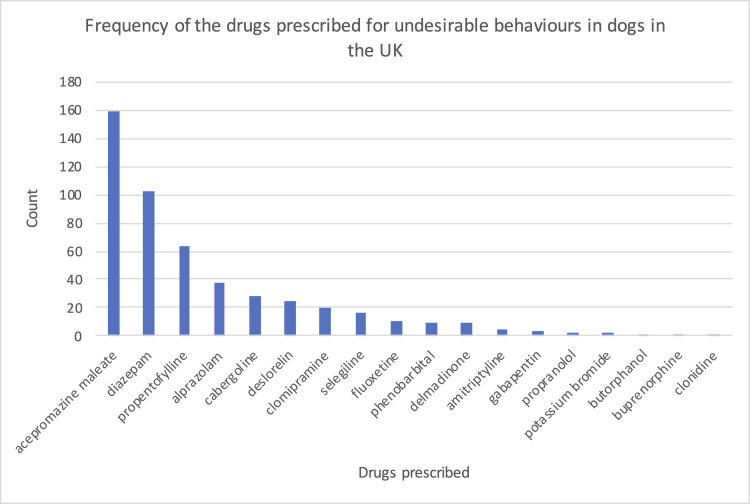
The frequency (count) of the drugs prescribed for UBs in dogs attending primary-care practices in the VetCompass™ Programme in the UK during 2013 (n = 496).

There were 496 prescribing events for drugs to treat the 413 UBs. Of these, 342 (82.8%) UBs were treated with one drug, 60 (14.5%) were treated with two drugs and 11 (2.7%) were treated with three or more drugs. Of the 496 prescribing events, the most frequently prescribed drugs for UB were acepromazine maleate (159; 32.1%), diazepam (102; 20.6%) and propentofylline (64; 12.9%) (Fig 5).

When broken down by age, acepromazine maleate was the most common drug prescribed to dogs of all age categories up to 12 (years), ranging from 32.6% in dogs aged 6 to < 9 to 42.3% in dogs aged 3 to < 6. Conversely, propentofylline was the most common drug prescribed to dogs ≥ 12 years, accounting for 39.9% drug-prescribed UBs in this age bracket (Table 2).

**Table 2:** The total number of prescribing events and three most commonly prescribed drugs by age group (count; %) in dogs attending primary-care veterinary practices in the VetCompass™ Programme in the UK during 2013 (n = 496).

Age (years)	Total number (%) of prescribing events	Most frequently prescribed drugs by age group (count; %)
< 3	81 (16.3%)	Acepromazine maleate (32; 39.5)
		Deslorelin (14; 17.3)
		Cabergoline (13; 16.1)
3 to < 6	111 (22.4%)	Acepromazine maleate (47; 42.3)
		Diazepam (20; 18.0)
		Alprazolam (8; 7.2)
6 to < 9	92 (18.6%)	Acepromazine maleate (30; 32.6)
		Diazepam (23; 25.0)
		Alprazolam (17; 18.5)
9 to < 12	79 (15.9%)	Acepromazine maleate (29; 36.7)
		Diazepam (22; 27.9)
		Propentofylline (9; 11.4)
≥ 12	133 (26.8%)	Propentofylline (53; 39.9)
		Diazepam (28; 21.1)
		Acepromazine maleate (21; 15.8)

There were 81/404 (20.1%) case dogs neutered within the available EPRs. Of these, 38/81 (46.9%) were neutered prior to the onset of a drug-prescribed UB and 43/81 (53.1%) were neutered after. Among those neutered afterwards, management of an UB was the main recorded reason stated for neutering in 13 (30.2%) dogs, prevention or treatment of another disorder in 8 (18.6%) dogs, owner convenience in 1 (2.3%) dog, with the reason not stated in the remaining 21 (48.8%) dogs. There were 9/404 (2.2%) dogs referred to a behaviourist during the study period. At a grouped-level of clinical description, the most common drug-prescribed UBs in the referred subset were aggression (4; 44.4%), anxious/distressed (3; 33.3%), noise phobia (1; 11.1%) and behaviour disorder (1; 11.1%). There were 73/404 (18.1%) deaths among the dogs with drug-prescribed UBs during the study period. The mechanism of death was not recorded in 7 (1.7%) cases. Of those with information recorded, 64/66 (97.0%) were euthanased and 2/66 (3.0%) died unassisted. An UB was reported to contribute to euthanasia in 31/64 (48.4%) deaths. There were 1/31 (0.03%) of UB-related euthanasia cases referred to a veterinary behaviourist before euthanasia.

### Risk factors for drug-prescribed UBs

All tested variables, other than *purebred*, were liberally (p < 0.2) associated with UB in univariable logistic regression modelling and were further evaluated in the main breed-based multivariable logistic regression modelling. The final main multivariable model retained five risk factors: *breed*, *age*, *sex-neuter*, *insurance* and *practice group* (Table 3). After accounting for the effects of the other variables evaluated, 3 breeds showed increased odds of drug-prescribed UB compared with crossbred dogs. The breeds with the highest odds were the Toy Poodle (OR 2.8, 95% CI 1.10 to 6.86, p = 0.030), Tibetan Terrier (OR 2.7, 95% CI 1.16 to 6.22, p = 0.022) and Shih-tzu (OR 2.0, 95% CI 1.10 to 3.46, p = 0.022) (Table 3). No breeds had reduced risk of drug-prescribed UB compared with crossbred dogs. Increased age was associated with increased odds of drug-prescribed UB, with dogs ≥ 12 years at 3.1 times the odds (95% CI 2.29 to 4.27, p < 0.001) compared with dogs < 3 years. Neutered males (OR 1.8, 95% CI 1.47 to 2.26, p <0.001) and entire males (OR 1.5, 95% CI 1.09 to 2.07, p = 0.013) had higher odds than entire females. Insured dogs had 1.8 (95% CI 1.47 to 2.26, p < 0.001) times the odds of drug-prescribed UB compared with uninsured dogs (Table 3). The Hosmer-Lemeshow test indicated acceptable model fit (p = 0.686) and the area under ROC curve (0.688) indicated moderate predictive ability.

**Table 3:** Final multivariable model for risk factors associated with drug-prescribed UBs in dogs under primary veterinary care in the UK during 2013 (n = 103,597).

Variable	Category	Odds Ratio	95% CI*	Category P-value	Variable P-value
Breed	Crossbreed	Base			0.013
	Toy Poodle	2.8	1.10 to 6.86	0.030	
	Tibetan Terrier	2.7	1.16 to 6.22	0.022	
	Shih-tzu	2.0	1.10 to 3.46	0.022	
	Golden Retriever	1.5	0.82 to 2.58	0.205	
	Rottweiler	1.4	0.57 to 3.50	0.462	
	Staffordshire Bull Terrier	1.3	0.83 to 1.92	0.277	
	Jack Russell Terrier	1.1	0.73 to 1.69	0.637	
	Border Collie	1.0	0.54 to 1.83	0.985	
	Pug	1.0	0.31 to 3.11	0.970	
	German Shepherd Dog	0.9	0.49 to 1.73	0.784	
	English Springer Spaniel	0.9	0.41 to 1.77	0.670	
	Cocker Spaniel	0.8	0.46 to 1.46	0.504	
	Yorkshire Terrier	0.8	0.43 to 1.53	0.524	
	West Highland White Terrier	0.8	0.42 to 1.51	0.491	
	Bichon Frise	0.8	0.27 to 2.05	0.570	
	Labrador Retriever	0.7	0.41 to 1.01	0.056	
	Border Terrier	0.6	0.18 to 1.82	0.342	
	Cavalier King Charles Spaniel	0.4	0.15 to 1.13	0.085	
	Short-Haired Chihuahua	0.3	0.04 to 2.07	0.215	
	Miniature Schnauzer	0.3	0.03 to 1.77	0.163	
	Boxer	0.2	0.03 to 1.35	0.097	
	Purebred—Other	1.1	0.83 to 1.49	0.477	
Age (years)	< 3	Base			< 0.001
	3 to < 6	1.1	0.78 to 1.48	0.658	
	6 to < 9	1.0	0.74 to 1.46	0.828	
	9 to < 12	1.5	1.06 to 2.10	0.023	
	≥ 12	3.1	2.29 to 4.27	< 0.001	
Sex-Neuter status	Female entire	Base			0.001
	Female neutered	1.3	0.93 to 1.85	0.120	
	Male entire	1.5	1.09 to 2.07	0.013	
	Male neutered	1.8	1.47 to 2.26	< 0.001	
Insurance	Non–insured	Base			< 0.001
	Insured	1.8	1.47 to 2.26	< 0.001	
Practice Group	1	Base			< 0.001
	2	1.8	1.16 to 2.83	0.010	
	3	1.6	0.97 to 2.56	0.064	
	4	2.2	1.50 to 3.27	< 0.001	

*95% Confidence Interval.

As described in the Methods, *adult bodyweight* individually replaced the *breed* variable in the final multivariable model but was not significant in the final multivariable model.

## Discussion

### Proportion of dogs with at least one UB event that received drug therapy

The estimated one-year period prevalence for dogs with at least one drug-prescribed UB was 0.4%. Given that previous studies have estimated that over 95% of dogs are perceived by their owners as performing UBs [5, 6], the current results suggest that only a small proportion of these dogs receive veterinary drug therapy for UBs in primary care practice. This may be for a number of reasons, related to the owner, veterinarian and dog. Owners may be unwilling or unable to use behaviour modifying drugs for their dogs’ UBs for a number of reasons, including lack of awareness of the welfare-relevance of their dog’s UBs (and thus the need for treatment) [19], reluctance to admit the UB is an issue or lack of awareness of psychopharmaceutical treatment possibilities for UBs. Other practical barriers may include owners finding regular oral administration of drugs more difficult than behaviour modifying products in diffuser or spray formats, e.g. DAP (Ceva Santé Animale) [19], and financial costs of regular treatment [32].

Considering other stakeholders in the treatment of UBs, it is possible that veterinarians in first opinion practice do not feel comfortable in providing behavioural support, which could include the prescription of behaviour modifying drugs for UBs. Previous studies have reported that veterinarians vary in their confidence in giving behavioural advice [63] and only 25% of veterinarians are reported to enquire about their patients’ behaviour regularly [64]. As a result, it may be that veterinarians suggest non-therapeutic options or choose to refer their patients to a behavioural specialist rather than trialling behaviour modifying drugs in first opinion practice, neither of which was the focus of the current study.

Turning to the dogs being treated, it is possible that many of the UBs which were reported to their veterinarians were minor, and their owners or veterinarian felt they did not warrant the use of drug therapy. In addition, it is possible that the reported UBs were found to be caused by other health problems, which were detected by their veterinarian, and treatment of which also treated the UB. A study in the USA recently reported that 15% of dogs presented with an UB for veterinary care had an underlying medical problem that potentially contributed to the UB [65]. The role of pain-related health problems as a cause of UBs is increasingly reported, including noise phobias [66] and aggression [67].

### Most common drug-prescribed UBs clinically described

At a general, grouped-level of clinical description, the two UBs most frequently reported were anxious/distressed (33.4%) and aggressive (18.6%). However, previous research suggests aggression is the most commonly reported UB in dogs referred to behaviourists [10, 12]. As aggression is an UB that can be highly hazardous to humans [64], it may have led to some dogs being euthanased or relinquished before drug therapy could be trialled. This is supported by previous epidemiological studies of causes of mortality in dogs aged three years and under, which revealed that 33.7% of dogs in the UK [11] and 29.7% in Australia [25] had UBs, with the most common UB reported as a cause of death being aggression (54% in England and 52.5% in Australia). Furthermore, within these studies, pharmaceutical, pheromone or nutraceutical treatments for aggression were attempted in just 3% of the UK sample [11] and 5.9% of the Australian sample [25].

Fear [32] and anxiety [30] represent natural canine responses to anxiogenic circumstances, but owners may vary in their consideration of these behaviours as undesirable [43]. Survey data indicates that UK dog owners rate aggression towards family members as the behaviour most likely to be considered a problem (56%), followed by destructive behaviour (51%) and house soiling (53%) [6]. Novice owners are more likely to report UBs than those who have owned dogs previously [43]. The purpose for which the dog was acquired may also influence the desirability of specific behaviours; for example, vocalisation may be encouraged in a dog obtained for property protection but be deemed undesirable in a dog obtained as a child’s pet [68]. Owners may also be more likely to report a behaviour as undesirable when it compromises their lifestyle or causes public shame [43]. As such, more attention should be directed towards these aspects of human psychology when treating UBs in dogs and attempting to educate their owners.

### Clinical management and outcomes of drug-prescribed UB cases

The most common drug used to treat UBs in the current study was acepromazine maleate (32.1%). This finding is somewhat surprising, as the use of sedatives to treat conditions such as phobias had been discouraged for 12 years at the time of the study (2013) [43]. It is possible that a study looking at more recent clinical records would show different results. Acepromazine has been used historically for the management of UBs by producing sedation and therefore reducing reactivity to environmental stimuli that trigger the UB [69]. The use of acepromazine as an anxiolytic can often have disappointing results and even cause other undesirable side-effects [70]. Benzodiazepines or serotonin reuptake inhibitors (SRIs) are now preferentially recommended [69]. The second most common drug used to treat UBs in the current study was diazepam (20.6%). A retrospective study of dogs prescribed diazepam for UBs by the Veterinary Hospital of the University of Pennsylvania found most owners reported its effectiveness as very (24%) or somewhat (43%) effective [71]. At the time of interview, 49% of owners were still administering diazepam to their dogs. However, for the remainder, 51% of owners reported discontinued use of diazepam due to adverse side effects (58%) (such as aggression, sedation and ataxia) and/or lack of efficacy (53%) [71]. Benzodiazepines can diminish conditioned responses and produce memory deficits, and consequently may compromise behavioural modification that relies on learning [69]. Furthermore, benzodiazepines may disinhibit behaviour and so should be used with caution in aggressive animals, as bite inhibition may decline [69].

Although the effectiveness of SRIs in conjunction with behaviour modification to reduce UBs has been noted in multiple studies [29, 31, 72–74], the current results reveal only 4.0% and 2.2% of behaviour modifying drugs prescribed were clomipramine and fluoxetine, respectively. The use of fluoxetine in combination with behavioural modification can assist in the management of inter-dog aggression [74] and SRBs [73, 75]. Similarly, clomipramine has been shown to improve signs of SRBs (urination, defecation and destruction) three times faster when used in combination with behavioural programs than the use of behavioural programs alone [72]. Additionally, unlike benzodiazepines, SRIs are unlikely to disinhibit behaviour [69] and therefore it might be expected that a greater number of dogs would have been prescribed these in the current study. The findings suggest that veterinarians prescribing behaviour modifying drugs for UBs may benefit from further education on the welfare benefits of the use of SRIs in conjunction with behaviour modification in preference to sedatives. However, it is important to note that the current study reflects prescribing behaviours by UK veterinarians in 2013 and could differ from prescribing behaviours outside of the UK and in more recent clinical notes.

Only 9/404 (2.2%) dogs that received UB drug therapy in the practice were referred to a behaviourist during the study period, this included dogs of any age. This could be considered cause for concern because successful resolution of UBs often requires skilled behavioural assessment and the use of behavioural and environmental modification strategies, alongside behaviour modifying drugs where appropriate [26]. Although, the current study did not investigate the overall prevalence of UBs for each individual within the given population and it is possible many may have received behavioural support or non-therapeutics that managed the UB effectively therefore rendering the use of UB drug therapy unnecessary. It is also possible that first opinion veterinarians may have felt comfortable prescribing UB drug therapy for those UB cases they felt competent to handle (such as a noise phobia) but may have escalated others to a specialist (or in extreme cases, such as aggression, euthanise) without the use of UB drug therapy. However, two previous VetCompass™ studies found only 10.3% [11] and 11.0% [25] of dogs under 3 years who died due to an UB were recommended a referral before their death in the UK and Australia, respectively. The current study also found an UB was reported to contribute to euthanasia in 31/64 (48.4%) dogs and only 1/31 of these dogs (0.03%) was referred to a veterinary behaviourist before euthanasia. This is relatively high and suggests that there is currently a missed opportunity for behaviour referral and potentially the use of behaviour modifying drugs with attempted management before resorting to euthanasia.

### Risk factors for drug-prescribed UBs

Failing to support our hypothesis that smaller bodyweight would be a risk factor for drug therapy aimed at managing UBs, the results of the current study showed that bodyweight was not a significant factor for the odds of receiving drug therapy related to an UB. Despite this, the 3 breeds at higher odds of an UB treated with a behaviour modifying drug (the Toy Poodle, Tibetan Terrier and the Shih-tzu) all have an average bodyweight of under 11kg [76]. It is possible that there is a subset of smaller breeds, rather than all smaller breeds, who are at an increased likelihood of being prescribed drug therapy for UBs. Differences in behavioural traits among different breeds, such as reactivity, aggressiveness and problem-solving ability [77, 78] could influence the relative prescription of behaviour modifying drugs. Many studies that link an inverse relationship between UBs and body size rely on owners’ reports of UBs [36, 37]. It has been proposed that UBs are more likely to be tolerated by owners in small dogs than large ones [37, 79] and that smaller dogs may be perceived by their owners as more aggressive, excitable, anxious, fearful and disobedient [79]. However, another study, where an inverse relationship between UBs and body size was also reported, relied upon the reports of trained observers and yielded very similar results [38]. It is also possible that these links reflect environmental influences in that different sizes of dog are often handled differently, not least in terms of physical restraint [11]. Furthermore, for the first opinion UK veterinary caseload under 3 years of age, there is evidence that dogs weighing over 40kg are less likely to die as a result of an UB than those in the lower weight categories [11]. It is possible that the owners of larger breeds, crosses and types invest in training to avoid or address UBs and reduce the risk of injuries [11].

Increasing age in the current study was associated with an increased odds of a drug-prescribed UB. A UK study reported that age in dogs was negatively correlated with the number of UBs performed and younger dogs were more likely to demonstrate SRB and UBs related to owner control (poor recall, stealing food, chasing things and pulling on the lead) [6]. Another US study found the severity and frequency of mouthing behaviour also negatively correlated with age [80]. As such, we would expect younger dogs to have increased odds of a drug-prescribed UB, which is in contrast to our results. It is possible that younger dogs may be less likely to be prescribed behaviour modifying drugs by veterinarians or have the recommendation accepted by owners. However, at the most precise level of clinical description, cognitive dysfunction was the second most common drug-prescribed UB (10.4%) and propentofylline the most common drug prescribed to dogs ≥ 12 years (39.9%). This suggests the inclusion of cognitive dysfunction may have increased the number of dogs with a drug-prescribed UB in the older age categories in this study, as by definition cognitive dysfunction affects aging dogs [81] and propentofylline can be used to treat dullness and lethargy in older dogs [82].

Previous VetCompass™ studies have stressed the importance of including insurance status within epidemiological studies to account for confounding [83] and the current results support this argument. Insured dogs had 1.8 times the odds of drug-prescribed UB compared with uninsured dogs. Because of reduced financial constraints, insured animals are more likely to receive veterinary care and to undergo diagnostic procedures than uninsured animals [84]. It is also possible that the differing costs of behaviour modifying drugs affected the decision of which ones were prescribed by veterinarians and accepted by owners.

Although some studies show no association between sex and the distribution of reported UB [6], others have reported that male dogs are at higher risk than females [8, 85]. Testosterone is thought to play a large role in an individual developing marking and howling when dogs are left alone [36]. The current results also suggest a male predisposition to UBs, with both neutered (OR 1.8) and entire males (OR 1.5) showing greater odds of a drug-prescribed UB than entire females. A previous study identified male dogs aged under 3 years with 1.4 times the odds of death due to UB than females [11]. It is possible that the higher risk of males performing UBs resulted in the increased likelihood of being prescribed a behaviour modifying drug, regardless of neuter status. It is also possible that the type of UBs that males demonstrate are considered as more amenable for pharmaceutical therapy. Previous research into the effects of neuter status on the likeliness of a dog demonstrating UBs is equivocal. Entire dogs are considered less likely to exhibit SRB [86] but more likely to exhibit aggressive behaviour [87] than neutered dogs. Conversely, other studies suggest that neutered individuals of both sexes have a greater probability of exhibiting aggression [88], while others suggest neuter status has no significant effect on the frequency or type of UB [6]. During the current study period, 20.1% of dogs prescribed a behaviour-modifying drug for an UB were neutered. Of those neutered after the UB event, management of the UB was the main reason stated for neutering in 30.2%. However, the evidence base supporting neutering as an effective modifier of UB is weak. In Norway (where routine neutering of dogs is discouraged), the most common reason cited by owners who did opt to neuter was the hope of reducing UBs (65%) but only 11.3% reported that the desired effect on their behaviour had been achieved [89]. Currently, there is little evidence that neutering dogs that demonstrate UBs unrelated to sexual behaviour (such as anxiety and fearfulness) is likely to produce a positive result [89].

## Limitations

This study had some limitations. Veterinarians often record clinical signs in lieu of formal diagnostic terms and this tendency was especially apparent for patients with UBs. This meant that the current study extracted clinical descriptions rather than formal diagnostic terms for many of the UBs. This resulted in the UBs being categorised in a number of different ways, including behavioural descriptions e.g. inappropriate toileting or excessive vocalisation, emotional assessments e.g. anxious/distressed, some specific clinical diagnoses e.g. cognitive dysfunction and noise phobia and the use of some other terms such as “aggressive” that potentially cover a variety of different behaviours and behavioural motivations. This is not surprising, as there is some debate about the most appropriate way to categorise behavioural problems in animals even amongst veterinary behavioural specialists [90, 91]. However, this inconsistency in how the UBs were categorised, coupled with the fact that there is no way of assessing the accuracy of the behavioural assessments made by the primary-care veterinarians, means that it is challenging to infer deeply about individual types or categories of UBs from this study.

This study may have under-represented the proportion of dogs with an UB that was managed with drug therapy, as clinical notes that lacked a clinical description of an UB directly linked to the prescription of the drug were not included in the study. The current study did not collect how many cases may have potentially been excluded by the need to have a prescription linked to an UB in the medical record and this may be a useful area for future research on UB drug therapy. Any of the reported outcomes may have also been under-represented for the same reason. For example, it may be that the veterinarian did not record if the owner was offered a referral and declined or contacted a behaviourist of their own accord.

This study reported dogs as cases provided they had at least one drug-prescribed UB in 2013 regardless of duration of administration. It is possible many of the drugs were prescribed for singular, acute events (e.g. Bonfire Night), and thus dominates the dispensation load because of this. Further research should explore differences in acute versus long term behaviour drugs for UBs. A study looking at more recent clinical notes might also be expected to show differences in the specific drugs prescribed and possibly also in the frequency of which drugs were prescribed for animals with UBs. The current study provides a useful baseline and the potential to see if any advances have been made in the use of drug therapy for management of UBs in the primary care setting since 2013.

Epilepsy and seizure disorders are generally considered neurological conditions, rather than behavioural, so they were excluded from the case definition [48]. That said, behavioural and cognitive comorbidities of canine epilepsy are increasingly recognised, including cognitive impairments [92–95], attention-deficit related behaviours [96, 97] and anxiety-related behaviours before onset [98, 99]. Some of these negative behavioural changes may be attributable to anti-seizure medications e.g. levetiracetam [100]. Therefore, idiopathic epilepsy and the use of anti-seizure drug treatment may be associated with the presence of UBs, and further research to understand the use of psychopharmacology in this complex population are needed.

## Conclusion

The estimated one-year period prevalence in dogs prescribed drug therapy related to an UB overall was 0.4%, which is markedly lower than previously published estimates of the prevalence of UBs overall. This study has described demographic risk factors for drug-prescribed UB as well as highlighted that UB was reported to contribute to euthanasia in 48.4% of dogs euthanased with drug-prescribed UBs. Furthermore, it has demonstrated very few dogs (2.2%) prescribed drug therapy for an UB were referred to a behavioural specialist during the study period. To improve the welfare of dogs, it is vital to improve veterinary expertise in the prevention and management of UBs in the primary care setting, but to also encourage referrals to behavioural specialists where appropriate, to optimise the quality of life of dogs with UBs, and their caregivers.

## Supporting information

S1 Table Behaviour modifying drugs included within the study and their corresponding search ‘stems’ and legal category. Prescription Only Medicine -Veterinarian (POM-V) may only be supplied to the client once it has been prescribed by a veterinary surgeon. Prescription Only Medicine—Controlled Drug (POM-CD) are listed in one of five Schedules in the Misuse of Drugs Regulations 2001 and the Misuse of Drugs Regulations (Northern Ireland) 2002. Off-license drugs are being used outside of the terms of their marketing authorisation. Note: only a limited number of brand names and their corresponding ‘stems’ are included within the table due to sizing constraints.
(DOCX)
