# Supplementary material for: Veterinary drug therapies used for undesirable behaviours in UK dogs under primary veterinary care
Source: PLoS One. 2022 Jan 12;17(1):e0261139. doi: 10.1371/journal.pone.0261139 (PMC8754320; doi:10.1371/journal.pone.0261139)
Supplement: S1 Table — Prescription Only Medicine -Veterinarian (POM-V) may only be supplied to the client once it has been prescribed by a veterinary surgeon. Prescription Only Medicine—Controlled Drug (POM-CD) are listed in one of five Schedules in the Misuse of Drugs Regulations 2001 and the Misuse of Drugs Regulations (Northern Ireland) 2002. Off-license drugs are being used outside of the terms of their marketing authorisation. Note: only a limited number of brand names and their corresponding ‘stems’ are included within the table due to sizing constraints. (DOCX) [file pone.0261139.s001.docx]

| Chemical name | Legal category | Code - Chemical name | Brand names 1 | Code - Brand names 1 |
| --- | --- | --- | --- | --- |
| 5-Hydroxy-L-tryptophan | GSL | GSL | 5-HTP | GSL |
| Acepromazine maleate | POM-V | Aceprom | ACP | ACP |
| Aktivait | GSL | GSL | DHA | GSL |
| Alpha-casozepine | GSL | GSL | Zylkene | GSL |
| Alprazolam | Off-Licence | Alpra | Xanax | Xanax |
| Amitriptyline | Off-Licence | Amitri |  |  |
| Buspirone | Off-Licence | Busp | Buspar | Busp |
| Cabergoline | POM-V | Caber | Galastop | Galas |
| Carbamazepine | Off-Licence | Carbama | Tegretol | Tegre |
| Chlordiazepoxide | Off-Licence | Chlordiaz |  |  |
| Citalopram | Off-Licence | Citalo | Celexa | Celexa |
| Clomipramine | POM-V | Clomi | Anafranil | Anafr |
| Clonazepam | Off-Licence | Clonaz | Klonopin | Klono |
| Clonidine | Off-Licence | Clonid | Catapres | Catapr |
| Clorazepate | Off-Licence | Cloraze | Tranxene | Tranx |
| Cyproheptadine | Off-Licence | Cyprohe | Periactin | Periac |
| Delmadinone | POM-V | Delma | Tardak | Tardak |
| Desipramine | Off-Licence | Desipr | Desmethylimipramine | Desmeth |
| Deslorelin | POM-V | Deslore | Suprelorin | suprel |
| Dextroamphetamine | Off-Licence | Dextroa | Dexedrine | Dexe |
| Diazepam | Off-Licence | Diaze | Valium | Valiu |
| Diphenhydramine | Off-Licence | Diphenh | Dreemon | Dree |
| Dog Appeasing Pheromone | GSL | GSL | Appeasines | GSL |
| Doxepin | Off-Licence | Doxe | Sinepin | Sinep |
| Duloxetine | Off-Licence | Dulox | Cymbalta | Cymba |
| Escitalopram | Off-Licence | Escita | Lexapro | Lexap |
| Flumazenil | Off-Licence | Flumaz | Anexate | Anexat |
| Fluoxetine | Off-Licence | Fluox | Prozac | Proz |
| Flurazepam | Off-Licence | Fluraz | Dalmane | Dalman |
| Fluvoxamine | Off-Licence | Fluvo | Fluvoxamine | Fluvo |
| Gabapentin | Off-Licence | Gabapenti | Gabapentinum | Gabapenti |
| Haloperidol | Off-Licence | Halope | Haldol | Haldo |
| Harmonease | GSL | GSL | Harmonease | GSL |
| Hydrocodone | Off-Licence | Hydrocodone | Zohydro ER | Zohydro |
| Hydroxyzine | Off-Licence | Hydroxyz | Atarax | Atarax |
| Imipramine | Off-Licence | Imipr |  |  |
| L-Theanine | GSL | GSL | Calmex | GSL |
| L-Tryptophan | GSL | GSL | Tryptan | GSL |
| Lithium salts | Off-Licence | Lithium | Camcolit | Camco |
| Lorazepam | Off-Licence | Loraz | Ativan | Ativa |
| Megestrol acetate | POM-V | Megest | Ovarid | Ovarid |
| Melatonin | GSL | GSL | Melatonin | GSL |
| Memantine | Off-Licence | Meman | Namenda | Namenda |
| Methylphenidate | Off-Licence | Methylphenidate | Concerta | Concerta |
| Midazolam | Off-Licence | Midazolam | Dormicum | Dormic |
| Mirtazepine | Off-Licence | Mirtaze | Zispin | Zisp |
| Naloxone | Off-Licence | Nalox | Narcan | Narc |
| Naltrexone | Off-Licence | Naltrex | Low Dose Naltrexone (LDN) | Naltrex |
| Nefazodone | Off-Licence | Nefazo | Dutonin | Duton |
| Nicergoline | POM-V | Nicerg | Fitergol | Fiterg |
| Nortryptiline | ? | Nortr | Pamelor | Pamelo |
| Oxazepam | POM CD | Oxaze |  |  |
| Paroxetine | Off-Licence | Paroxe | Seroxat | Serox |
| Pentazocine | POM CD | Pentaz | Talwin | Talw |
| Phenobarbital | POM-V, POM CD | Phenobarb | Phenobarbitone | Phenobarb |
| Pindolol | Off-Licence | Pindo | Visken | Visk |
| Propentofylline | POM-V | Propento | Vivitonin | Vivitonin |
| Propranolol | Off-Licence | Propranol | Inderal | Indera |
| Protriptyline | Off-Licence | Protrip | Vivactil | Vivact |
| Pyridoxine | GSL | GSL | Vitamin B6 | GSL |
| Reboxetine | Off-Licence | Rebox | Edronax | Edrona |
| S-Adenosylmethionine (SAMe) | GSL | GSL | Zentonil Plus | GSL |
| Selegiline | Off-Licence | Seleg | L-Deprenyl | Depren |
| Sertraline | Off-Licence | Sertra | Lustral | Lustral |
| Temazepam | Off-Licence | Temaz | Restoril | Restori |
| Trazodone | Off-Licence | Trazod | Molipaxin | Molipa |
| Venlafaxine | Off-Licence | Venlaf | Efexor | Efex |

|  |  |
| --- | --- |
